# Effect of Different Blood-Pressure-Lowering Regimens on the Blood Pressure Control among Hypertensive Patients Treated in Hospital Conditions

**DOI:** 10.1155/2020/3097198

**Published:** 2020-10-21

**Authors:** Anna Paczkowska, Karolina Hoffmann, Krzysztof Kus, Dorota Kopciuch, Tomasz Zaprutko, Piotr Ratajczak, Elżbieta Nowakowska, Wiesław Bryl

**Affiliations:** ^1^Department of Pharmacoeconomics and Social Pharmacy, Poznan University of Medical Sciences, Poznań, Poland; ^2^Department of Internal Diseases, Metabolic Disorders and Arterial Hypertension, Poznan University of Medical Sciences, Poznań, Poland

## Abstract

**Background:**

Scientific references lack sufficient amount of data on analyses of the reasons for hospital admissions or assessment of efficacy of arterial hypertension treatment at hospitals.

**Objectives:**

The aim of the study was to evaluate the efficacy of antihypertensive drug therapy on the blood pressure control among hospitalized hypertensive patients. *Methodology*. A cross-sectional retrospective study consisted of 204 patients aged 18–65 years admitted to the hospital due to hypertension between January 2018 and December 2018. The study was based on analysis of electronic records, obtained from the medical database of the selected healthcare facility.

**Results:**

As a result of the treatment applied at the hospital, 65.19% of the patients achieved the desired degree of blood pressure normalization (≤130/80 mmHg). Vast majority of the patients during their stay at the ward would receive three or more hypertensive drugs (63.73%). The most frequently prescribed antihypertensive drug combinations included bitherapies such as diuretics + ACEI and ACEI + *β*-blockers and tritherapy such as diuretics + *β*-blockers and calcium channel antagonists and diuretics + ACEI and ARBs. The highest blood-pressure lowering effects were observed among patients receiving combination therapy of a ACEI, a diuretic, and a ARBs. Tritherapy induced a significant mean reduction of inpatients`s SBP compared with bitherapy (*p*=0.0001).

**Conclusion:**

During their hospital stay, vast majority of patients (65.19%) achieved normal values of blood pressure, mostly owing to combined treatment with several hypertensive drugs. Efficacy of the most frequently used combinations of hypertensive drugs in normalizing arterial pressure varies.

## 1. Introduction

Arterial hypertension (AH) remains the most modifiable risk factor for cardiovascular disease, and according to the World Health Organization (WHO), it is still the first cause of premature death in the world. It is estimated that 7.5mln people die every year due hypertension complications, making up 12.8% of all deaths worldwide [1]. According to the latest ESC/ESH guidelines (European Society of Cardiology/European Society of Hypertension) from 2018, hypertension can be diagnosed if the average BP values (calculated from at least two measurements made during at least two different visits) are equal or higher than 140 mm Hg for SBP and/or 90 mm Hg for DBP [2]. According to the American guidelines of the American Heart Association/American College of Cardiology 2017 (AHA/ACC 2017) and European guidelines of ESC/ESH 2018, optimal reduction of the global risk of cardiovascular complications is obtained in younger patients (<65 y. o.) by lowering BP to less than 130/80 mm Hg in most patients with AH, including patients with associated ischemic heart disease, after having had a heart attack or stroke [2]. Data obtained from long-term observation of the subjects of the first large clinical trials (Systolic Hypertension in Elderly Program, SHEP) [3] show that one month of hypertensive therapy extends the patient's life by one day. In spite of documented benefits and availability of a safe and effective therapy, insufficient control of the population's blood pressure remains a widespread problem. According to NATPOL 2011 study's results, the desired degree of blood pressure control (below 140/90 mmHg) is achieved in 26% of patients with hypertension in Poland [4]. As a result of the failure to normalize their blood pressure values, many patients require hospital treatment. In 2014, 7.2 mln people were hospitalized in Poland, of which 14.2% had been admitted due to cardiovascular diseases [5]. Costs of hospital treatment of hypertension account for 7% of overall treatment costs. In 2010, average per patient cost of hospitalization due to hypertension amounted to EUR 357 [6]. Professional references lack any analyses on the reasons for hospital admissions or assessment of efficacy of hypertension treatment at hospitals.

## 2. Objective

The aim of the study was to analyze the reasons for hospital admission and evaluate the efficacy of antihypertensive drug therapy on the blood pressure control among hypertensive patients treated in hospital conditions.

## 3. Methodology

### 3.1. Study Population

The retrospective cross-sectional survey was conducted at the leading provincial clinic of hypertension treatment in Poland over the time of one year (from 01/01/2018 to 12/31/2018). Based on the available data from the selected healthcare facility's medical database and on the adopted inclusion and exclusion criteria, 204 patients aged 18–65 years, suffering from hypertension, were qualified for assessment of efficacy of hypertension treatment at hospitals.

Inclusion criteria in the study were as follows:

- The patient's age between 18 and 65 years
- Diagnosed and treated hypertension
- Admission to hospital as a result of the lack of control of blood pressure despite outpatient pharmacotherapy
- Urgent admission to hospital as a result of high blood pressure

Exclusion criteria in the study were as follows:

- The patient's age under 18 and above 65 years
- Pending diagnosis of hypertension

### 3.2. Study Technique

The present cross-sectional retrospective survey relied on analysis of medical records of hospitalized hypertensive patients. Data collected from these medical records concerned the sex, age, duration of the diseases, admission mode, any comorbidities, blood pressure values at admission to and discharge from the ward, average daily blood pressure values, type of pharmacotherapy used, and recommendations given to patients upon discharge from the hospital. Evaluation of the efficacy of hypertension treatment in hospital environment was based on the patient's medical data, percentage of patients whose blood pressure values were normalized with the medical procedures and pharmacotherapy type used, and based on average daily blood pressure values (24-Hour Ambulatory Blood Pressure Monitoring) of the patients during hospital treatment. In accordance with the latest guidelines of the American Heart Association/American College of Cardiology 2017 (AHA/ACC 2017) and European guidelines, ESC/ESH 2018 values below 130/80 mm Hg were adopted as target blood pressure values [2]. In addition, as a result of the analysis of available patients' medical records, a comparative analysis of the effectiveness of blood pressure normalization of the four most commonly used combinations of antihypertensive drugs among the examined patients was performed. As a criterion for the effectiveness of blood pressure normalization of selected hypertension treatment regimens, the difference in blood pressure values upon admission to the hospital and upon discharge from the hospital was adopted as well as the percentage of patients who achieved blood pressure targets in a given therapeutic group. The research project was approved by the management of the hospital to which the patients had been admitted due to hypertension and received a positive opinion from the Local Bioethics Committee.

### 3.3. Statistical Analysis

The collected data were analyzed using MS Excel sheets. The chi-squared (c^2^) test, Mann–Whitney *U* test, Kruskal–Wallis test on ranks (ANOVA), and post-hoc test of multiple comparisons of mean rank packages for all samples were also used. Spearman's rank correlation was also calculated. Results were identified as statistically significant at the significance level of *p*=0.05. Statistica 7.1 package by StatSoft was used.

## 4. Results

In 2018, 1,218 patients were admitted to the Internal Diseases, Metabolic Disorders, and Hypertension Ward. Based on the adopted study inclusion and exclusion criteria, 204 patients aged 18–65 years admitted to the hospital due to lack of control of blood pressure despite outpatient pharmacotherapy and urgent admission to hospital as a result of high blood pressure were qualified for assessment of efficacy of hypertension treatment at hospitals. Most of the patients admitted due to hypertensive disorder (69.61%) were male. Average age of the study subjects was 41.77 ± 14.53 years. The largest percentage of patients (29.41%) admitted to the hospital due to hypertension were aged 50–65 years. Analysis of the available medical records leads to a conclusion that the most common comorbidities in the study group of patients suffering from hypertension include obesity (54.41%), type 2 diabetes (42.64%), angina pectoris (12.25%), heart failure (8.82%), and previous brain stroke (5.88%) (Table 1).

Most of the patients (73.53%) were admitted to the ward urgently, while 26.47% of admissions were planned. Urgent admissions were applied to patients with elevated systolic blood pressure values (*p* < 0.001). According to the analysis of the records, the largest number of patients (62.75%) were those referred to the hospital due to primary (essential) hypertension identified in the International Classification of Diseases (ICD10) with code I10. The remaining patients had the following diseases diagnosed upon admission: hypertensive disorder affecting the heart, without heart failure (23.53%) hypertensive disorder affecting the heart, with heart failure (8.82%), secondary hypertension (7.84%), and resistant hypertension (1.96%) (Table 2).

Systolic blood pressure values upon admission to the hospital amounted to 168.92 mmHg ± 15.05 mmHg, while diastolic blood pressure values 109.25 mmHg ± 14.47 mmHg. The Mann–Whitney *U* test with a significance rate of *p*=0.049 has shown statistically significant differences in terms of systolic blood pressure between sexes. Women had a higher systolic pressure. Systolic blood pressure values upon discharge from the hospital amounted to 128.00 mmHg ± 15.14 mmHg, while diastolic blood pressure values 78.17 mmHg ± 10.32 mmHg. Average daily systolic blood pressure values during hospitalization amounted to 128.54 mmHg ± 14.53 mmHg, while diastolic blood pressure values were 75.77 mmHg ± 8.64 mmHg. Statistical analysis using the Mann–Whitney *U* test with significance rate of *p*=0.031 has shown statistically significant differences in terms of average daily systolic blood pressure value between sexes. Men had higher average daily systolic pressure values. No statistically significant differences between sexes were found in systolic or diastolic blood pressure values upon admission to or discharge from the hospital (Table 3).

Average length of stay at the hospital ward was 7.52 ± 3.05 days. The Mann–Whitney *U* test has shown that the elevated blood pressure values of women who stayed at the hospital ward for normalization were statistically significantly longer (*p*=0.001) than those for men (8.48 ± 3.47 vs 7.10 ± 2.75 days). A positive correlation has also been shown between the subjects' age and length of hospital stay (R Spearman = 0.159, *p*=0.022). The older the subject, the longer their stay at the hospital was.

Analysis of medical records has shown that the number of hypertensive drugs used during hospitalization in the study group was 3.18 ± 1.47 on average. Vast majority of the patients during their stay at the ward would receive three or more hypertensive drugs (63.73%). 23.04% of the patients were treated with two hypertensive drugs while monotherapy was administered to 13.23% of the patients admitted to the hospital due to hypertension. The Mann–Whitney *U* test has shown that, during hospitalization, men would be receiving a significantly larger number of hypertensive drugs than women (3.32 ± 1.49 vs 2.87 ± 1.36, *p*=0.049). In addition to this, statistical analysis using Spearman's rank correlation has shown a correlation between the number of hypertensive drugs administered during the subjects' hospital stay and their age and average daily values of systolic and diastolic blood pressure values. The older the subjects and the higher their average daily values of systolic and diastolic blood pressure values were, the greater the number of hypertensive drugs received during their hospital stay would be (*p*=0.001).

Analysis of medical records has found that, during hypertension treatment at the hospital, 87.75% were receiving diuretics, 69.60%–*β*-blockers, 63.81%–ARBs, 61.27%–ACEI, 53.43%–calcium channel antagonist, and 5.39%–a fixed*-*dose combination drug. Of diuretics, the most frequently used active ingredients included indapamide (26.47% of the subjects) and torasemide (38.23%), and of *β*-blockers, bisoprolol (28.92%) and carvedilol (16.17%). Of hypertensive drugs from the ARBs group, the most frequently used were walsartan (36.17% of the subjects) and telmisartan (11.54%), of calcium channel antagonists, amlodipine (45.58%), and of fixed*-*dose combination drugs, perindopril combined with amlodipine (19.60%) (Table 4).

Eighty-four patients (41.17%) received acetylsalicylic acid, while 70.58% had statins introduced into their therapy during their hospital stay. 17.64% of the patients required potassium and magnesium supplementation. Due to a large percentage of patients with type 2 diabetes, 45.58% of the patients received oral antidiabetic agents.

As a result of the treatment applied at the hospital, 65.19% of the patients achieved the desired degree of blood pressure normalization (≤130/80 mmHg).), including 35.29% with blood pressure values below ≤120/70 mmHg.

Detailed analysis of pharmacotherapy administered to hospitalized hypertensive patients has shown that 19 patients (9.3%) received pharmacological treatment in the form of a combination of two hypertensive drugs, a *β*-blocker and an ACEI. Same percent of patients (9.3%) received pharmacological treatment in the form of a combination of two hypertensive drugs, a diuretic and an ACEI. A large portion of the study group (26.47%) during their hospital stay would receive pharmacotherapy based on a combination of three hypertensive drugs: a *β*-blockers, a diuretic, and a calcium channel antagonist. Another most frequently recorded polytherapy was a combination of an ACEI, a diuretic, and a ARB, administered to 25 patients (12.25%).

Statistical analysis performed has shown statistically significant differences among comparative analysis of the most frequently used combinations of hypertensive drugs in terms of lowering blood pressure values during the hospital stay. The highest blood-pressure-lowering effects were observed among patients receiving combination therapy of an ACEI, a diuretic, and an ARBs. Tritherapy induced a significant mean reduction of inpatients`s SBP compared with bitherapy (*p*=0.0001). The percentage of hospitalized patients with controlled BP was the highest among patients receiving biotherapy based on a combination of a *ß*-blockers + an ACEI (87.50% patients achieved the desired degree of blood pressure normalization-≤130/80 mmHg). Nevertheless, in this group of patients, the average systolic and diastolic blood pressure upon admission to the hospital was the lowest among compared blood-pressure-lowering regimens. Statistically significant differences were found in the efficacy of hypertension treatment using bitherapy based on a combination of a *ß*-blockers + an ACEI and a bitherapy based on a combination of a diuretic and an ACEI. In group of patients receiving *ß*-blockers + an ACEI, the average systolic blood pressure values upon discharge from the hospital was significantly lower compared with the group of patients receiving a diuretic and an ACEI (*p* < 0.0001) (Table 5).

Statistically significant differences were found in efficacy of hypertension treatment using tritherapy based on a combination of *β*-blockers + diuretics + calcium channel antagonist and a tritherapy based on a combination of a diuretic, an ACEI, and an ARBs. In group of patients receiving *β*-blockers + diuretics + calcium channel antagonist, the average systolic and diastolic blood pressure values upon discharge from the hospital were significantly lower compared with the group of patients receiving a diuretic, an ACEI, and an ARBs (*p* < 0.0001) (Table 5).

## 5. Discussion

In spite of progress in diagnostics, identification, and treatment of hypertensive disorder, only in 60% of the patients, the therapy manages to reduce blood pressure values to less than 140/90 mmHg [7]. Results of many studies [8, 9] clearly indicate that poorer medication adherence is associated with poor BP control and a higher risk of cardiovascular diseases and all-cause hospitalization in hypertensive patients. This study has shown that among patients admitted to the hospital due to hypertension, 23% had a diagnosed hypertensive disorder affecting the heart, without heart failure, 8.82% had a hypertensive disorder affecting the heart, with heart failure, and 5.88% of the patients were admitted following a brain stroke.

The largest percentage of patients (29.41%) admitted to the hospital due to arterial hypertension were aged 60–80 years. 69.61% of the hospitalized patients were male. Most of the patients (73.53%) were admitted to the ward urgently, while 26.47% of admissions were planned. The main cause for hospital admission was high blood pressure among patients with a diagnosed primary (essential) hypertension (62.75%). These results are corroborated in the studies by other authors. According to the study by Bachórzewska-Gajewska et al. [10] to analyze the causes of hospital admissions and type of treatment administered to hypertensive patients, most of the subjects (80%) were admitted to hospitals urgently, and more than a half of them were aged above 55 years. Similarly to our study, most of the patients (78.9%) admitted to the hospital due to hypertension 23% had a diagnosed primary hypertension while 17.8% had a diagnosed hypertensive disorder affecting the heart.

Our study found that average systolic blood pressure values upon admission to the hospital amounted to 168.92 mmHg ± 15.05 mmHg while diastolic blood pressure values to 109.25 mmHg ± 14.47 mmHg. Women had statistically significantly higher systolic blood pressure values upon admission. As a result of the treatment applied at the hospital, 65.19% of the patients achieved the desired degree of blood pressure normalization (≤130/80 mmHg), while 35.29% of the patients had blood pressure values of ≤120/70 mmHg upon discharge from the hospital. These results corroborate with the studies by other authors [10–12].

According to the available references, most patients in order to normalize their elevated blood pressure values require a polytherapy based on a combination of three or more hypertensive drugs [10, 11, 13]. In our study, more than 63% of the patients received a combination therapy with three or more hypertensive drugs. 23.04% of the patients were treated with two hypertensive drugs while monotherapy was administered to 13.23% of the patients admitted to the hospital due to hypertension. For many years, monotherapy and gradual increases of drug doses has been the recommended mode of treatment, but results of multiple clinical trials proved the efficacy of monotherapy to be limited [14]. According to ESH/ESC, achievement of the desired blood pressure level frequently requires polytherapy, specifically in people with a high cardiovascular risk whose blood pressure values significantly exceed the threshold values [13]. This results from the fact that pathogenesis of hypertension is a very complex mechanism dependent on many factors. Moreover, effect of individual ingredients of the therapeutic combination on various mechanisms responsible for blood pressure growth has additional benefits. It entails a significantly better tolerance profile by mutual neutralization of the drugs' adverse effects, contributing to better compliance [15, 16]. Reinforcing the above, several clinical studies have shown that patients receiving more antihypertensive drugs achieved (as expected) lower blood pressure figures and had greater reductions in the appearance of cardiovascular and cerebrovascular events [17–19].

Detailed analysis of pharmacotherapy administered to patients admitted to the hospital due to high blood pressure has shown that a largest number of patients were receiving diuretics (87.75%), *β*-blockers (69.60%), ARBs (63.81%), ACEI (61.27%), calcium channel antagonists (53.43%), and fix-dose combination drugs (5.39%). These results corroborate with those of the studies by other authors. In the study by Potchoo et al. [11] evaluating the effect of antihypertensive drug therapy on blood pressure control among hospitalized and ambulatory hypertensive patients, diuretics were the most frequently prescribed drugs either as a single agent or as combination therapy with other classes of antihypertensive drugs. The combinations consist of at least one diuretic controlled blood pressure in 97.14% vs 87.50% of ambulatory and hospitalized patients, respectively. ESH recommendations clearly emphasize that hypertensive therapy should be tailored to every single patient's case. When choosing the drug, the managing physician should take into account a number of factors such as hypertension severity, the patient's age, occupation and preferences, financial resources, effect of the drug on other risk factors of cardiovascular diseases, presence of complications in the organs, and the risk of occurrence of potential drug interactions and adverse effects [12, 13, 19].

Detailed analysis of polytherapy administered to patients admitted to the hospital due to high blood pressure has shown that the largest number of patients were receiving the following combinations of hypertensive drugs: a *ß*-blocker in combination with an ACEI (9.3%), an ACEI in combination with a diuretic (9.3%), a *ß*-blocker in combination with a diuretic and a calcium channel antagonist (26.47%), and an ACEI in combination with a diuretic and a ARBs (12.25%). Moreover, statistical analysis performed has shown a statistically significant differences among comparative analysis of the most frequently used combinations of hypertensive drugs in terms of lowering blood pressure values during the hospital stay. The highest blood-pressure-lowering effects were observed among patients receiving combination therapy of an ACEI, a diuretic, and an ARBs. Tritherapy induced a significant mean reduction of inpatients`s SBP compared with bitherapy. These results are corroborated in other research papers [11, 20–22]. In the study by Potchoo et al. [11], 39.13% of the hospitalized hypertensive patients received bitherapy, 33.33% tritherapy, 20.29% quadritherapy, and 7.25% monotherapy. Combination therapy was administered to 92.75% of the patients. The most frequently prescribed antihypertensive drug combinations included bitherapies such as diuretics + ACEI and diuretics + calcium channel antagonists, tritherapy with diuretics + ACEI and calcium channel antagonists and quadritherapy with diuretics + ACEI, and calcium channel antagonists and centrally acting antihypertensive drug. Quadritherapy induced a significant mean reduction of inpatients' SBP compared with monotherapy and with biotherapy. The combinations including at least one diuretic induced a significant reduction of inpatients' SBP. Multiple clinical and observational studies have also reported clinically relevant differences among antihypertensive drugs, in terms of both BP lowering efficacy and tolerability/safety profile [22]. These differences should be taken into account not only when adopting first-line antihypertensive therapy but also when titrating or modulating combination therapies, with the aim of achieving effective and sustained BP control. Most combinations of antihypertensive agents, whether at fixed doses or free combinations, include a diuretic. These combinations have been shown to produce greater blood pressure reductions than those seen with monotherapies [18]. Law et al. [17] concluded that the effects of combination medications were additive. In addition, the combination medications responded better than either medication used alone. For instance, the combination of ACE inhibitor and thiazide diuretic was shown to have advantages over the two monodrug therapies. The ACE inhibitors block the counterregulatory increase in the angiotensin II triggered by diuretic therapy; conversely, thiazide diuretics may stimulate the rennin-angiotensin system and enhance the antihypertensive action of ACE inhibitors.

### 5.1. Study Strong Points

Our studies are innovative as scientific references lack sufficient amount of data on analyses of the reasons for hospital admissions or assessment of efficacy of arterial hypertension treatment at hospitals. Moreover, no research papers to date have performed a comparative analysis to assess the efficacy of the most frequently used combinations of hypertensive drugs (bitherapy and tritherapy) in normalizing elevated blood pressure values. Available research data only directly compare combinations of antihypertensive drugs based on biotherapy [23, 24]. The results of our research provide scientific evidence for clinicians and health care decision-makers to create new standards in the treatment of hypertension. Our results proved the efficacy of monotherapy to be limited. Based on the results of the research, specialist and primary care physicians will be aware of which of the most frequently used forms of polytherapy in clinical practice of arterial hypertension are the most effective in normalizing blood pressure.

### 5.2. Study Limitations

Our study has some limitations though. The most important limitation is the fact that this study sample was recruited from a single center. The study population was relatively small (*n* = 204) and may be difficult to be generalized. It would be very interesting to roll the study out to other centres afterwards. In addition, in the comparative analysis of the effectiveness of the most commonly used forms of polytherapy in the normalization of blood pressure, we limited the comparative analysis only in relation to hypertensive drug groups, without taking into account active substances and drug doses, which was due to the small number of the studied group. Considering insufficient number of studies related to analyses of the reasons for hospital admissions and assessment of efficacy of arterial hypertension treatment at hospitals in Poland however, this study might be recognized as an important contribution in the field.

## 6. Conclusions

Based on the study conducted, it must be concluded that arterial hypertension patients are most frequently admitted to hospitals due to inefficacy of outpatient treatment and complications of their hypertensive disorder. During their hospital stay, vast majority of patients (65.19%) achieved normal values of arterial pressure, mostly owing to combined treatment with several hypertensive drugs. Efficacy of the most frequently used combinations of hypertensive drugs in normalizing arterial pressure varies. Hypertensive therapy should be individualized.

## Figures and Tables

**Table 1:** General characteristics of hospitalized patients with arterial hypertension (*n* = 204).

Group size	Total	204
	Female	62
	Male	142

Age (years)	Total $\bar{x}$ ± (SD)^*∗*^	56.77 ± 14.53
	Female $\bar{x}$ ± (SD)	59.74 ± 14.13
	Male $\bar{x}$ ± (SD)	55 ± 14.56

Body mass index, BMI (kg/m^2^)	Total $\bar{x}$ ± (SD)^*∗*^	34.26 ± 8.02
	Female $\bar{x}$ ± (SD)	33.84 ± 7.86
	Male $\bar{x}$ ± (SD)	34.45 ± 8.12

Duration of the disease (years)	Total $\bar{x}$ ± (SD)^*∗*^	11.06 ± 8.26
	Female $\bar{x}$ ± (SD)	11.49 ± 7.96
	Male $\bar{x}$ ± (SD)	10.87 ± 8.41

Most common comorbidities	Obesity	54.41%
	Diabetes type 2	42.64%
	Angina pectoris	12.25%
	Heart failure	8.82%
	Brain stroke	5.88%

$\bar{x}$ (SD)^*∗*^: average (standard deviation).

**Table 2:** ICD classification of the patients admitted to the hospital due to hypertension (*n* = 204).

Disease code and name	*n*	%
I10, Essential (primary) hypertension	128	62.74
I11.9, Hypertensive heart disease without heart failure	48	23.53
I15, Secondary hypertension-renovascular hypertension	19	9.31
I11, Hypertensive heart disease with heart failure	8	3.92
I12.9, Hypertensive chronic kidney disease without renal failure	1	0.49

ICD: International Classification of Diseases.

**Table 3:** Blood pressure values of the patients admitted to the hospital due to hypertension (*n* = 204).

Blood pressure measurement	Systolic blood pressure values (mmHg)	Diastolic blood pressure values (mmHg)
Upon admission to the hospital	168.92 ± 15.05	109.25 ± 14.47
Upon discharge from the hospital	128.00 ± 15.14	78.17 ± 10.32
Daily average values (ABPM)	128.54 ± 14.53	75.77 ± 8.64

ABPM: ambulatory blood pressure monitoring.

**Table 4:** Hypertensive drugs used by the patients admitted to the hospital due to hypertension (*n* = 204).

Hypertensive drugs group	Active substance	Percentage of patients receiving the drug (%)
Angiotensin-converting enzyme inhibitors (ACEI)	Overall	61.27
	Ramipril	25.98
	Captopril	19.11
	Perindopril	7.84
	Verapamil	2.45
	Chinapril	5.88

*β*-Blockers	Overall	69.30
	Bisoprolol	28.92
	Carvedilol	16.17
	Nebivolol	16.17
	Metoprolol	8.33

Diuretics	Overall	87.75
	Indapamid	26.47
	Furosemid	5.39
	Torasemid	38.23
	Spironolactone	17.64

Calcium channel antagonist	Overall	53.43
	Amlodipine	45.58
	Lercanidipine	7.84

Angiotensin II receptor antagonists (ARBs)	Overall	30.88
	Valsartan	17.64
	Telmisartan	5.39
	Losartan	6.37
	Candesartan	1.47

*α*-Adrenolytics	Overall	28.43
	Doxasosin	23.52
	Clonidine	4.90

Fixed*-*dose combination drug	Overall	5.39
	Perindopril + amlodipine	2.45
	Valsartan + amlodipine	1.96
	Ramipril + amlodipine	0.98

**Table 5:** Evaluation of efficacy of hypertension treatment in hospital environment according to the most common pharmacotherapy regimens.

Pharmacotherapy regimen	Number of patients on the pharmacotherapy regimen	Blood pressure measurement upon admission to the hospital		Blood pressure measurement upon discharge from the hospital		Blood pressure control (<130/80 mmHg) %
		Average systolic blood pressure values (mmHg, SEM ± SD)	Average diastolic blood pressure values (mmHg, SEM ± SD)	Average systolic blood pressure values (mmHg, SEM ± SD)	Average diastolic blood pressure values (mmHg, SEM ± SD)	
*β*-blockers + ACEI (A)	19 (9.3%)	156.26 ± 12.04	94.37 ± 7.83	122.37 ± 9.06 $*p*^*∗*^ < 0.001	73.63 ± 12.68 $*p* < 0.001	87.50 &*p*=0.0381 ^*p*=0.0006
Diuretics + ACEI (B)	19 (9.3%)	157.21 ± 9.46	92.68 ± 8.01	124.26 ± 9.42 $*p* < 0.001	76.79 ± 8.7 $*p* < 0.001	76.47 ^*p*=0.0077
*β*-blockers + diuretics + calcium channel antagonist (C)	54 (26.47%)	163.67 ± 14.12 ^*∗*^*p*=0.0454	94.61 ± 11.34	131.31 ± 12.30 ^*∗*^*p*=0.0049 #*p*=0.0261 $*p* < 0.0001	79.43 ± 6.97 ^*∗*^*p*=0.015 $*p* < 0.0001	61.81 ^*p*=0.0324
ACEI + diuretics + ARBs (D)	25 (12.25%)	182.00 ± 23.06 ^*∗*^*p* < 0.0001 #*p* < 0.0001 &*p* < 0.0001	103.32 ± 19.74 #*p*=0.0326 &*p*=0.0151	136.24 ± 16.25 ^*∗*^*p*=0.0018 #*p*=0.0065 $*p* < 0.0001	82.80 ± 8.95 ^*∗*^*p*=0.0074 #*p*=0.0311 $*p* < 0.0001	36.00

*p*: level of statistical significance; *p* < 0.05 was considered statistically significant. ^*∗*^Statistically significant value in relation to group A for *p* < 0.05. #: statistically significant value in relation to group B for *p* < 0.05. &: statistically significant value in relation to group C for *p* < 0.05. ^: statistically significant value in relation to group D for *p* < 0.05. $: statistically significant value in relation to SBP reduction and DBP reduction among antihypertensive class combinations.

## Data Availability

The data used to support the findings of this study are available from the corresponding author upon request.

## Abbreviations

**ABPM**: Ambulatory blood pressure monitoring
**ACEI**: Angiotensin converting enzyme inhibitors
**AH**: Arterial hypertension
**ARBs**: Angiotensin II receptor blockers
**BP**: Blood pressure
**DBP**: Diastolic blood pressure
**ESC/ESH**: (European Society of Cardiology/European Society of Hypertension)
**SBP**: Systolic blood pressure.
